# Open questions on basal insulin therapy in T2D: a Delphi consensus

**DOI:** 10.1007/s00592-024-02285-2

**Published:** 2024-05-20

**Authors:** Delphi Board, Aglialoro Alberto, Anichini Roberto, Avogaro Angelo, Baggiore Cristiana, Berra Cesare, Bonadonna Riccardo, Corrao Salvatore Maria Giuseppe, Da Porto Andrea, De Candia Lorenzo, De Cosmo Salvatore Alessandro, Di Cianni Graziano, Formoso Gloria, Garrapa Gabriella, Ghiani Mariangela, Giorgino Francesco, Guaita Giacomo, Maiorino Maria Ida, Masi Stefano, Modugno Monica, Morea Nicola, Morviducci Lelio, Napoli Nicola, Napoli Raffaele, Occhipinti Margherita, Orsi Emanuela, Perseghin Gianluca, Piro Salvatore, Sartore Giovanni, Sesti Giorgio, Tassone Francesco, Trevisan Roberto, Raffaella Buzzetti, Riccardo Candido, Katherine Esposito, Andrea Giaccari, Edoardo Mannucci, Antonio Nicolucci, Giuseppina T. Russo

**Affiliations:** 1 Department of Experimental Medicine, Sapienza University of Rome, Rome, Italy; 2 Department of Medical Surgical and Health Sciences, University of Trieste, Trieste, Italy; 3 Department of Advanced Medical and Surgical Sciences, Università della Campania “Luigi Vanvitelli”, Naples, Italy; 4 Center for Endocrine and Metabolic Diseases, Fondazione Policlinico Universitario A. Gemelli IRCCS and Università Cattolica del Sacro Cuore, Rome, Italy; 5 Diabetology, Careggi Hospital and University of Florence, Florence, Italy; 6 CORESEARCH – Center for Outcomes Research and Clinical Epidemiology, Corso Umberto I, 103, 65122 Pescara, Italy; 7 Department of Clinical and Experimental Medicine, University of Messina, Messina, Italy

**Keywords:** Type 2 diabetes, Expert consensus, Basal insulin therapy, Therapeutic inertia

## Abstract

**Aims:**

The revolution in the therapeutic approach to type 2 diabetes (T2D) requires a rethinking of the positioning of basal insulin (BI) therapy. Given the considerable number of open questions, a group of experts was convened with the aim of providing, through a Delphi consensus method, practical guidance for doctors.

**Methods:**

A group of 6 experts developed a series of 29 statements on: the role of metabolic control in light of the most recent guidelines; BI intensification strategies: (1) add-on versus switch; (2) inertia in starting and titrating; (3) free versus fixed ratio combination; basal-bolus intensification and de-intensification strategies; second generation analogues of BI (2BI). A panel of 31 diabetologists, by accessing a dedicated website, assigned each statement a relevance score on a 9-point scale. The RAND/UCLA Appropriateness Method was adopted to assess the existence of disagreement among participants.

**Results:**

Panelists showed agreement for all 29 statements, of which 26 were considered relevant, one was considered not relevant and two were of uncertain relevance.

Panelists agreed that the availability of new classes of drugs often allows the postponement of BI and the simplification of therapy. It remains essential to promptly initiate and titrate BI when required. BI should always, unless contraindicated, be started in addition to, and not as a replacement, for ongoing treatments with cardiorenal benefits. 2BIs should be preferred for their pharmacological profile, greater ease of self-titration and flexibility of administration.

**Conclusion:**

In a continuously evolving scenario, BI therapy still represents an important option in the management of T2D patients.

## Introduction

Insulin is useful and valuable to maintain glycemic control once progression of the disease overcomes the effect of other glucose-lowering agents [1]. Thus, many adults with type 2 diabetes (T2D) eventually require and benefit from insulin therapy. However, many issues surround the position of insulin therapy in T2D management algorithm.

With the availability of new classes of glucose-lowering drugs, particularly GLP-1 RA and SGLT2i, with good efficacy, cardiorenal protection and acceptable side effect profiles, the initiation of insulin has been postponed in many patients to later stages of the disease. In particular, the consensus report by ADA and EASD recommend GLP-1 RA as first injectable medication for patients with atherosclerotic cardiovascular disease or indicators of high risk [2]. Furthermore, if an injectable therapy is needed to reduce HbA1c, a GLP-1 RA should be considered in most patients prior to insulin, as they allow lower glycemic targets to be reached with a lower injection burden and lower risk of hypoglycemia and weight gain than with insulin alone [1, 2]. However, many patients still need therapy intensification with insulin, in addition to, or in substitution of other ongoing treatments. In this regard, the combination of basal insulin (BI) and GLP-1 RA has potent glucose lowering actions and is associated with less weight gain and hypoglycemia compared with fully intensified insulin regimens [1], thus representing a valid alternative to multiple daily injections of insulin.

At variance with existing guidelines, real world data show that the initiation of insulin therapy and its intensification is often considerably delayed, even though the patient has high blood glucose levels, remaining above target even for years [3]. Moreover, despite the complementary actions of GLP-1 RA and BI, real world data show that GLP-1 RA therapy is often discontinued when BI is introduced [4].

Guidelines emphasize that, in all insulin-treated people with T2D, agents associated with cardiorenal protection or weight reduction should be maintained in the treatment regimen whenever possible [1]. However, since routine clinical practice involves heterogeneous real-life patient populations, there remains uncertainty on how and when to use free or fixed ratio combination of BI and GLP-1 RA.

Additional issues regarding the treatment with insulin in everyday practice are represented by the choice of 1st versus 2nd-generation BI and the choice between free versus fixed-ratio combinations (FRC) of GLP-1 RA and BI.

For many years, the most widely used BI analog has been insulin glargine 100 U/mL, a first-generation analogue of basal insulin (1BI), whose efficacy and safety, including cardiovascular safety, are well established [5]. In most recent years, second-generation analogue of basal insulins (2BIs) [glargine 300 U/mL (Gla-300) and degludec 100 U/mL (IDeg-100)] have become available. EDITION and BEGIN registration programs documented that 2BIs provide similar or improved efficacy with a better safety profile compared to 1BIs [6], as a consequence of their improved pharmacokinetic/pharmacodynamic profiles [7]. However, most clinical practice guidelines do not explicitly state whether and under which circumstances 2BIs should be preferred to 1BIs. Recently, Italian guidelines have provided a strong recommendation to initiate or switch to 2BIs for all patients with T2D needing basal insulin therapy [13].

Finally, as people with T2D get older, it may become necessary to simplify complex insulin regimens due to a decline in self-management ability. Treatment simplification aims to decrease the complexity of treatment regimens, including, but not limited to fewer administration times and fewer blood glucose checks. Results of RCTs suggest that it is possible to switch from a basal bolus insulin regimen to a combination of BI plus either a GLP-1 RA or a SGLT2i, with same or better glycemic control, less injections, less insulin doses, less hypoglycemia and increased satisfaction of therapy [8]. Providing guidance to healthcare providers on how and when therapy simplification should be pursued thus represents an important issue.

Given the substantial number of open questions surrounding insulin treatment in T2D and the lack of solid scientific evidence to give an answer to all these issues, an expert panel was organized, with the aim of providing practical guidance to clinicians. To this purpose, a Delphi approach was used, involving representatives of the Italian diabetes societies. The process aimed at gathering experts’ opinions and eliciting consensus regarding the role of basal insulin in the treatment of patients with T2D, with particular focus on the role of metabolic control, the strategies to adopt for basal insulin intensification (timing, add-on vs. switch, free vs. fixed combinations), the strategies for intensification/de-intensification from basal-bolus treatment, and the role of second-generation analogues of basal insulins.

## Methods

### Development of statements

Statements were developed by a Steering group composed of six experts in diabetes management, members of the two main Italian diabetes societies (Società Italiana di Diabetologia—SID and Associazione Medici Diabetologi—AMD). In a face-to-face meeting, chaired by a panel moderator experienced in facilitating group discussions and criteria development, the experts were asked to identify key aspects of BI treatment in T2D relative to the following topics: role of metabolic control in light of the most recent guidelines; BI intensification strategies: add-on versus switch; BI intensification strategies: inertia to initiate and inertia to titrate; intensification strategies: free versus FRC; intensification and de-intensification strategies from basal-bolus; second-generation analogues of basal insulin. A total of 29 statements were identified and grouped in 6 main topics (Table 1).

**Table 1:** Results of Delphi process

	#	Statements	Median	30th percentile	70th percentile	IPRAS-IPR	Agreement	Decision
Role of metabolic control in the light of the most recent guidelines	1	Achieving the target of HbA1c < 7% has a significant impact on the prevention of microangiopathic complications	9	9	9	15.00	Yes	Relevant
	2	Achieving the target of HbA1c < 7% has a significant impact on the prevention of macroangiopathic complications	7	7	8	8.38	Yes	Relevant
	3	The suggested target of HbA1c between 6.6% and 7.5% is a goal that can be safely achieved in the majority of patients receiving second-generation basal insulin analogues, in the absence of other treatments that may cause hypoglycemia	8	8	8	11.25	Yes	Relevant
	4	Achievement of the HbA1c target has a secondary role in cardiorenal prevention	2	1	4.7	4.36	Yes	Irrelevant
Basal insulin intensification strategies: add-on versus switch	5	Insulin therapy should be considered at any stage of the natural history of the disease, if situations such as severe hyperglycemia, symptoms of glycemic decompensation or significant unintentional weight loss are present	9	8	9	12.13	Yes	Relevant
	6	The add-on of basal insulin therapy allows the achievement of optimal metabolic control in most patients on therapy with SGLT2i and/or GLP-1 RA not at target	8	7	8	8.38	Yes	Relevant
	7	The reduction in glucotoxicity achieved with basal insulin therapy improves the response to ongoing non-insulin therapies	7	7	8.7	8.99	Yes	Relevant
	8	In the patient starting basal insulin, it is not appropriate to suspend a pre-existing therapy with SGLT2i and/or GLP-1 RA, unless specific contraindications or tolerability problems are present	9	9	9	15.00	Yes	Relevant
	9	Switching from SGLT2i and/or GLP-1 RA to basal insulin is appropriate in case of specific clinical situations such as contraindications, tolerability issues, unwanted weight loss	8	7	8	8.38	Yes	Relevant
Basal insulin intensification strategies: inertia to initiate and inertia to titrate	10	Timely titration to an individualized glycemic target is necessary to obtain the full benefits of basal insulin	9	8	9	12.13	Yes	Relevant
	11	Inertia to initiate and titrate basal insulin to personalized glycemic target is often a cause of treatment failure	8	8	9	12.13	Yes	Relevant
	12	Self-titration of basal insulin should be recommended for all patients, except those who are unable to manage it	9	8	9	12.13	Yes	Relevant
Intensification strategies: free versus fixed combination	13	Basal insulin should be added to the GLP-1 RA only after the maximum tolerated dose of GLP1-RA has been reached	6.5	5	7	1.75	Yes	Uncertain
	14	Free combination of basal insulin and GLP-1 RA optimizes the effects of GLP-1 RA on cardiovascular risk, body weight, and glycemic control	8	8	9	12.13	Yes	Relevant
	15	Fixed-ratio combinations of basal insulin and GLP-1 RA rarely allow to achieve GLP-1 RA doses at which extra-glycemic benefits have been demonstrated	7	5	8	2.63	Yes	Relevant
	16	Fixed ratio combinations of basal insulin and GLP-1 RA do not allow for the optimal titration of individual molecules	7	6	8	5.50	Yes	Relevant
	17	Fixed-ratio combinations of basal insulin and GLP-1 RA represent a valid therapeutic strategy in patients showing side effects to GLP-1 RA, as they allow for a more gradual titration of the two drugs	6.5	6	8	5.50	Yes	Uncertain
Intensification strategies and de-intensification from basal-bolus in type 2 diabetes	18	In patients failing therapy with oral drugs (not GLP-1 RA) + basal insulin, intensification by adding GLP-1 RA to basal insulin, in fixed or free combination, may help achieve personalized HbA1c target	8	7	8	8.38	Yes	Relevant
	19	In patients failing therapy with oral drugs (not GLP-1 RA) + basal insulin, intensification by adding GLP-1 RA offers the advantage of the extra-glycemic benefits of this class of drugs	8	8	9	12.13	Yes	Relevant
	20	In patients treated with a basal-bolus insulin scheme, it is advisable to periodically reassess the effective need for this therapy	8	7.3	9	10.11	Yes	Relevant
	21	In patients treated with a basal-bolus insulin scheme, the addition of GLP-1 RA can allow a suspension of prandial boluses while maintaining glycemic control	8	7	8	8.38	Yes	Relevant
	22	In patients treated with a basal-bolus insulin scheme, the addition of GLP-1 RA can allow a suspension of prandial boluses, improving quality of life	8	7.3	8.7	9.85	Yes	Relevant
	23	Fixed-ratio combinations of basal insulin and GLP-1 RA can represent a therapeutic alternative for simplification and consequent de-intensification of therapy in patients treated with a basal-bolus insulin scheme	8	7	8	8.38	Yes	Relevant
Second-generation analogues of basal insulin	24	Second-generation basal insulin analogues are associated with a lower risk of hypoglycemia than previous formulations	9	9	9	15.00	Yes	Relevant
	25	The lower hypoglycemic risk of second-generation basal insulin analogues allows titration aimed at more stringent metabolic control compared to previous formulations	8	8	9	12.13	Yes	Relevant
	26	Second-generation basal insulin analogues allow easy and efficient titration of basal insulin by the patient	8	7	8	8.38	Yes	Relevant
	27	Flexibility in the timing of administration of second-generation basal insulin analogues compared to the usual timing allows easier achievement of glycemic targets	7	7	8.7	8.99	Yes	Relevant
	28	Flexibility in the timing of administration of second-generation basal insulin analogues allows for better therapeutic adherence	7.5	7	9	9.25	Yes	Relevant
	29	The pharmacokinetic and pharmacodynamic characteristics of second-generation basal insulin analogues allow them to be administered at different times of the day (e.g., in the morning)	9	8	9	12.13	Yes	Relevant

### Participants

Given the nature of the topic, the initiative only involved diabetes specialists, being the management of insulin therapy almost exclusively operated by diabetologists in Italy. A panel of 31 diabetologists was identified, selected on the basis of their long clinical and research experience in the field. Participants of both genders were sampled from different geographic areas and healthcare settings (university vs. non-university centers).

### Rating of statements

In June 2023, candidate panel members were invited by email to join the project and a web meeting was organized to explain the rationale and structure of the initiative. After acceptance, they were emailed personal credentials to access the dedicated website, containing the 29 statements identified by the Steering group, and asked to rate each on a nine-point scale. Ratings of 1–3 were classified as irrelevant, with a rating of one indicating the greatest degree of irrelevance. Ratings of 7–9 were classified as relevant, with a rating of nine indicating the greatest degree of relevance. Ratings of 4–6 were classified as neither relevant nor irrelevant.

Panel members were requested to make a short comment explaining the rationale for their rating to each statement, or to suggest rephrasing if the statement was ambiguous or not clear. After the end of the first round, results were tabulated.

Ratings of statements collected during the panel process were analyzed quantitatively to determine the existence of consensus among participants. As described in the RAND/UCLA Appropriateness Method, this process started with determining the existence of disagreement among participants using the following a priori process. First, we calculated the value of interpercentile range (IPR), or the range of responses that fell between the 70th and the 30th percentiles; second, we calculated the value of the interpercentile range adjusted for symmetry (IPRAS), which is a measure of dispersion for asymmetric distributions; finally, we compared the values of IPR and IPRAS to see if there was disagreement. Disagreement (lack of consensus) is said to exist if IPR > IPRAS [9].

Disagreement among participants automatically produced an uncertain decision. In the presence of an agreement among panelists, the value of the median obtained determined whether the specific statement was considered relevant, irrelevant, or uncertain. If the median fell within the upper tertile of the 9-point response scale (response categories 7–9), then the statement was considered relevant, meaning that the content of the statement is important in guiding clinical decision. If the median was within the lower tertile of the 9-point response scale (response categories 1–3), then the statement was considered irrelevant, meaning that its content was not useful to guide clinical practice. A median that lied within the middle tertile (response categories 4–6) produced an uncertain decision.

Following the assessment of consensus among participants, each panel member was provided with a copy of the frequency distribution of ratings of all panelists across the nine point scale, the overall panel median rating for each of the statements and an annotation of how they had rated each of the criteria. Scores from other panel members were not revealed. Depending on panelists votes, panel agreement or disagreement was also stated for each of the round one criteria. A second and third round were foreseen to facilitate consensus in case of statements for which disagreement was documented. However, agreement was reached at the first round for all the items, making additional evaluation by the panel unnecessary. Results of the first round were finally shared and discussed with all the 31 diabetologists through an ad hoc virtual meeting.

**Fig. 1:**
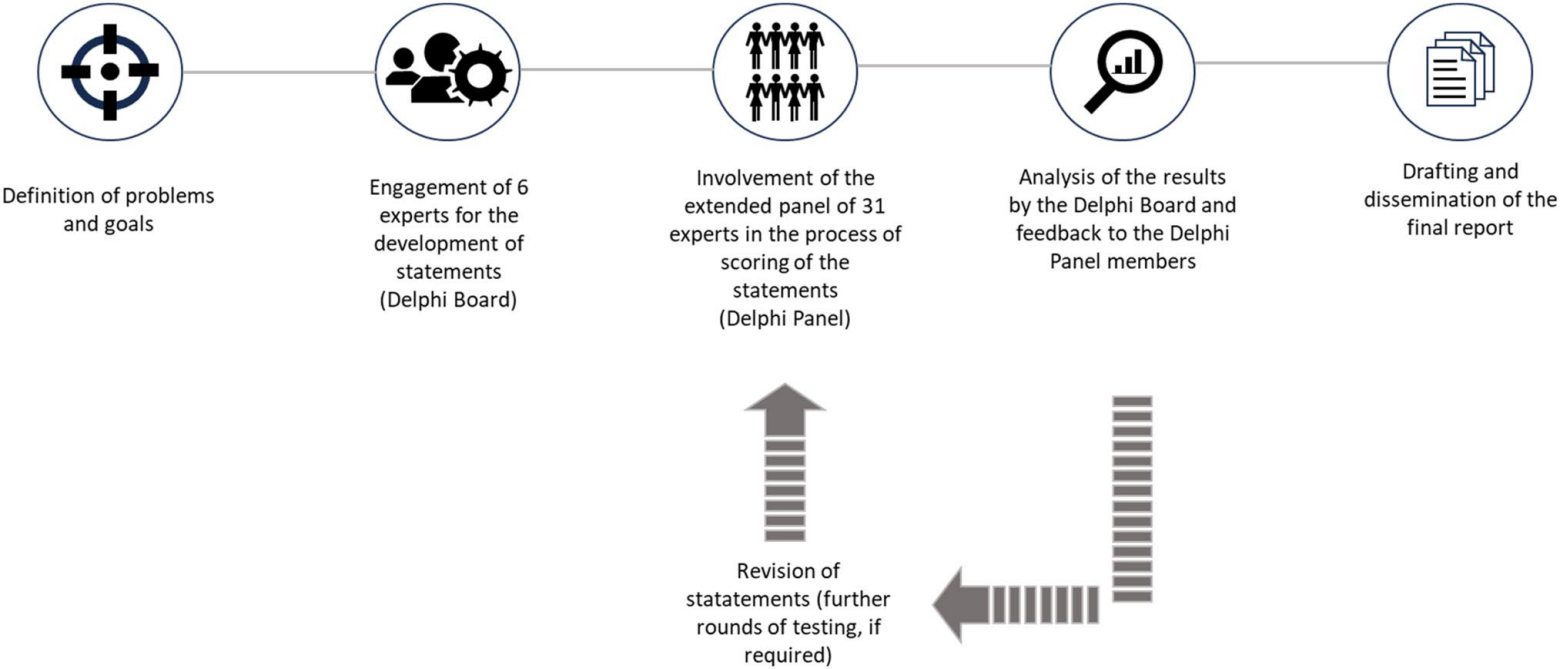
Structure of the DELPHI Consensus

The overall structure of the process is reported in Fig. 1.

## Results

All the 31 involved panelists responded to the questionnaire (response rate 100%). After completion of the first round, for none of the 29 statements panel members showed significant disagreement (IPR < IPRAS). Overall, 26 statements were considered as relevant, while for one item there was agreement that it was irrelevant. For two statements the relevance was uncertain. Table 1 reports the results relative to individual statements, while Tables 2 and 3 summarize respectively the major evidence gaps and statements with higher level of consensus emerging from the Delphi process.

**Table 2:** Evidence gaps emerging from the Delphi process (statements with score < 8)

Item #	Statement	Scoring	Comment
2	Achieving the target of HbA1c < 7% has a significant impact on the prevention of macroangiopathic complications	7	The statement is largely supported by current literature on the beneficial effects of reducing HbA1c on cardiovascular risk However, cardiovascular benefits are mitigated by the consequences of hypoglycemia, which is the counterbalance of tight glucose control. Furthermore, cardiovascular risk is multifactorial and needs to be addressed by an integrated approach, not limited to correcting hyperglycemia New evidence on the efficacy of multifactorial approach using more recent treatment options would be needed
7	The reduction in glucotoxicity achieved with basal insulin therapy improves the response to ongoing non-insulin therapies	7	The initiation of BI can improve the response to other ongoing glucose-lowering therapies through the reduction of glucotoxicity. However, the assumption is true for non-insulin drugs acting through the stimulation of insulin secretion, rendered ineffective by glucotoxicity, while it does not apply in the case of non-insulin drugs whose mechanism of action does not depend on the efficiency of the beta cell There is a lack of strong evidence supporting this statement
13	Basal insulin should be added to the GLP-1 RA only after the maximum tolerated dose of GLP1-RA has been reached	6.5	There was uncertainty as to whether BI should be added to the GLP-1 RA only after the maximum tolerated dose of GLP-1 RA has been reached. According to some of the experts, reaching the maximum tolerated dose of GLP-1 RA is essential to maximize cardiorenal benefits and minimize insulin doses, hypoglycemic events and weight gain. On the other hand, often BI is introduced before reaching the maximum tolerated dose of GLP-1 RA, particularly in case of poor metabolic control
15	Fixed-ratio combinations of basal insulin and GLP-1 RA rarely allow to achieve GLP-1 RA doses at which extra-glycemic benefits have been demonstrated	7	Free combinations have the advantage of potentially attain the maximum dosage of GLP-1 RA, thus maximizing its beneficial “dose-dependent” effects on cardiovascular protection as well as on glucose and body weight while avoiding the risk of “over-basalization” There is a lack of RCTs testing FRCs on major cardiorenal outcomes
16	Fixed-ratio combinations of basal insulin and GLP-1 RA do not allow for the optimal titration of individual molecules	7	FRC usually do not allow in clinical practice to reach the maximum dosage of GLP-1 RA, thus potentially limiting extraglycaemic effects. However, no RCT comparing FRC with free combination is available
17	Fixed-ratio combinations of basal insulin and GLP-1 RA represent a valid therapeutic strategy in patients showing side effects to GLP-1 RA, as they allow for a more gradual titration of the two drugs	6.5	The slow titration of a FRC has been suggested to increase the tolerability of GLP-1 receptor agonists compared with the use of GLP1-RA and basal insulin individually, reducing the occurrence and severity of gastrointestinal adverse events. However, additional evidence is needed to support this statement
27	Flexibility in the timing of administration of second-generation basal insulin analogues compared to the usual timing allows easier achievement of glycemic targets	7	In principle, flexibility in the timing of administration could facilitate titration and improve treatment adherence, particularly in patients with irregular lifestyle habits. However, solid evidence supporting this statement is lacking
28	Flexibility in the timing of administration of second-generation basal insulin analogues allows for better therapeutic adherence	7.5	Although there was agreement regarding the concept that flexibility in the timing of administration of second-generation BI analogues allowed for better therapeutic adherence, the consensus was mainly based on theoretical grounds and personal experience of participants

**Table 3:** Summary of the statements with high level of consensus (score ≥ 8)

**Role of metabolic control in light of the most recent guidelines**
- Achieving the target of HbA1c < 7% has a significant impact on the prevention of microangiopathic complications (Statement #1) - The suggested target of HbA1c between 6.6% and 7.5% is a goal that can be safely achieved in the majority of patients receiving second-generation basal insulin analogues, in the absence of other treatments that may cause hypoglycemia (Statement #3)
**Basal insulin intensification strategies: add-on versus switch**
- Insulin therapy should be considered at any stage of the natural history of the disease, if situations such as severe hyperglycemia, symptoms of glycemic decompensation or significant unintentional weight loss are present (Statemen #5) - The add-on of basal insulin therapy allows the achievement of optimal metabolic control in most patients on therapy with SGLT2i and/or GLP-1 RA not at target (Statement #6) - In the patient starting basal insulin, it is not appropriate to suspend a pre-existing therapy with SGLT2i and/or GLP-1 RA, unless specific contraindications or tolerability problems are present (Statement #8) - Switching from SGLT2i and/or GLP-1 RA to basal insulin is appropriate in case of specific clinical situations such as contraindications, tolerability issues, unwanted weight loss (Statement #9)
**Basal insulin intensification strategies: inertia to initiate and inertia to titrate**
- Timely titration to an individualized glycemic target is necessary to obtain the full benefits of basal insulin (Statement #10) - Inertia to initiate and titrate basal insulin to personalized glycemic target is often a cause of treatment failure (Statement #11) - Self-titration of basal insulin should be recommended for all patients, except those who are unable to manage it (Statement #12)
**Intensification strategies: free versus fixed combination**
- Free combination of basal insulin and GLP-1 RA optimizes the effects of GLP-1 RA on cardiovascular risk, body weight, and glycemic control (Statement #14)
**Intensification strategies and de-intensification from basal-bolus in type 2 diabetes**
- In patients failing therapy with oral drugs (not GLP-1 RA) + basal insulin, intensification by adding GLP-1 RA to basal insulin, in fixed or free combination, may help achieve personalized HbA1c target (Statement #18) - In patients failing therapy with oral drugs (not GLP-1 RA) + basal insulin, intensification by adding GLP-1 RA offers the advantage of the extra-glycemic benefits of this class of drugs (Statement #19) - In patients treated with a basal-bolus insulin scheme it is advisable to periodically reassess the effective need for this therapy (Statement #20) - In patients treated with a basal-bolus insulin scheme, the addition of GLP-1 RA can allow a suspension of prandial boluses while maintaining glycemic control (Statement #21) - In patients treated with a basal-bolus insulin scheme, the addition of GLP-1 RA can allow a suspension of prandial boluses, improving quality of life (Statement #22) - Fixed-ratio combinations of basal insulin and GLP-1 RA can represent a therapeutic alternative for simplification and consequent de-intensification of therapy in patients treated with a basal-bolus insulin scheme (Statement #23)
**Second-generation analogues of basal insulin**
- Second-generation basal insulin analogues are associated with a lower risk of hypoglycemia than previous formulations (Statement #24) - The lower hypoglycemic risk of second-generation basal insulin analogues allows titration aimed at more stringent metabolic control compared to previous formulations (Statemen #25) - Second-generation basal insulin analogues allow easy and efficient titration of basal insulin by the patient (Statement #26) - The pharmacokinetic and pharmacodynamic characteristics of second-generation basal insulin analogues allow them to be administered at different times of the day (e.g., in the morning) (Statement #29)

### Role of metabolic control in the light of the most recent guidelines

All the panelists totally agree (statement #1; median value of 9) that achieving the target of HbA1c < 7% has a significant impact on the prevention of microangiopathic complications, motivating their rating with the large amount of scientific evidence linking metabolic control with the risk of these complications. Also, a consensus was reached about the importance of achieving the target of HbA1c < 7% to prevent macrovascular complications (statement #2; median value of 7). A general agreement, with a median rating of 8, was reached regarding the possibility of safely achieving the suggested target of HbA1c between 6.6% and 7.5% in the majority of patients receiving second-generation BI analogues, in the absence of other treatments that may cause hypoglycemia (statement #3). Finally, panelists disagreed with the statement declaring that the achievement of the HbA1c target has a secondary role in cardiorenal prevention (statement #4; median rating 2). All the experts emphasized that strong evidence exists linking metabolic control to renal complications, although the effect on cardiovascular protection is still a matter of debate.

### Basal insulin intensification strategies: add-on versus switch

There was a strong consensus regarding the relevance of taking into consideration insulin therapy at any stage of the natural history of the disease, if situations such as severe hyperglycemia, symptoms of glycemic decompensation or significant unintentional weight loss are present (statement #5; median rating 9). In this respect, it was emphasized that, in the case of severe glyco-metabolic decompensation and hypercatabolism, optimized insulin therapy remains the therapy of choice, allowing for faster achievement of established glycemic goals. Also, panelists agreed that the add-on of BI allows the achievement of optimal metabolic control in most patients on therapy with SGLT2i and/or GLP-1 RA not at target (statement #6; rating 8), without losing the cardio-renal protection offered by GLP-1 RAs or SGLT2i.

A general agreement was reached, albeit with a lower level of relevance (statement #7; rating 7), regarding the possibility offered by the initiation of BI of improving the response to other ongoing glucose-lowering therapies through the reduction of glucotoxicity. In this respect, it was objected that the assumption is true for non-insulin drugs acting through the stimulation of insulin secretion, rendered ineffective by glucotoxicity, while it does not apply in the case of non-insulin drugs whose mechanism of action does not depend on the efficiency of the beta cell.

There was a strong consensus (statement #8; median rating 9) regarding the importance of not suspending a pre-existing therapy with SGLT2i and/or GLP-1 RA when starting BI, unless specific contraindications or tolerability problems are present. On the other hand, panelists agreed that a switch from SGLT2i and/or GLP-1 RA to BI is appropriate in case of specific clinical situations such as contraindications, tolerability issues, or unwanted weight loss with these drugs (statement #9; median rating 8).

### Basal insulin intensification strategies: inertia to initiate and inertia to titrate

There was a large consensus (statement #10; median rating 9) regarding the need to timely titrate BI to exploit the full benefits of therapy in the achievement of individualized glycemic targets. Panelists emphasized that trials with titration algorithms have shown that titration is essential to obtain the maximum benefit from this therapy. It was also commented that in clinical practice insulin therapy is frequently under-titrated. Experts convened that inertia to initiate and titrate BI to personalized glycemic target is often a cause of treatment failure (statement #11; median rating 8). Panelists emphasized the role of diabetes structured self-management education to involve patients in insulin titration and improve treatment adherence. On the same line, there was a strong agreement (statement #12; median rating 9) that self-titration of BI should be recommended for all patients, except those who are unable to manage it. Self-titration was considered a basic condition for the rapid achievement of the desired glycemic targets. The need to involve caregivers in educational activities was also emphasized.

### Intensification strategies: free versus fixed ratio combination

There was uncertainty as to whether BI should be added to the GLP-1 RA only after the maximum tolerated dose of GLP-1 RA has been reached (statement #13; median rating 6.5). There was agreement that the free combination of BI and GLP-1 RA optimizes the effects of GLP-1 RA on cardiovascular risk, body weight, and glycemic control (statement #14; median rating 8). It was observed that the free combination of BI and GLP-1 RA makes it possible to optimize the dosage of both molecules, allowing the administration of the maximum tolerated dose of GLP-1 RA and ensuring the related benefits on cardiovascular risk and body weight. The free combination also allows to maintain the maximum dosage of GLP-1 RA in patients who require small amounts of BI, thus reducing the risk of over-basalization. A general agreement was reached, albeit with a lower level of relevance (statement #15; median rating 7), regarding the statement that FRCs of BI and GLP-1 RA rarely allow to achieve GLP-1 RA doses at which extra-glycemic benefits have been demonstrated. On the same line, a consensus was reached, again with a lower level of relevance (statement #16; median rating 7) that FRCs do not allow for the optimal titration of individual molecules. Although some experts pointed out the difficulty in reaching the optimal dose of GLP-1 RA, others suggested that positive results can be obtained through an improvement in patient adherence and the simplification of the treatment.

Finally, there was uncertainty (statement #17; median rating 6.5) as to whether FRCs can represent a valid therapeutic strategy in patients showing side effects to GLP-1 RA, allowing for a more gradual titration of the two drugs.

### Intensification strategies and de-intensification from basal-bolus in type 2 diabetes

There was agreement that in patients failing therapy with oral drugs (other than oral GLP-1 RA) + BI, intensification by adding GLP-1 RA to BI, in fixed or free combination, may help achieve personalized HbA1c target (statement #18; median rating 8), and that this approach offers the advantage of the extra-glycemic benefits of GLP-1 RA (statement #19; median rating 8). In fact, the benefits of adding a GLP-1 RA to BI are clearly documented.

A consensus was also reached (statement #20; median rating 8) regarding the need, in patients treated with a basal-bolus insulin regimen, to periodically reassess the effective need for this therapy. It has been underlined that often this scheme is the “heritage” of a therapy set up during a hospital admission or an intercurrent illness, and then not re-evaluated. A periodical reassessment has also been suggested for obese patients who lose weight. Panelists also agreed that in patients treated with a basal-bolus insulin regimen, the addition of GLP-1 RA can allow a suspension of prandial insulin while maintaining glycemic control (statement #21; median rating 8) and improving quality of life (statement #22; median rating 8), as supported by existing evidence. There was also consensus that FRCs can represent a therapeutic alternative for simplification and consequent de-intensification of therapy in patients treated with a basal-bolus insulin regimen (statement #23; median rating 8). Reported arguments in favor of this approach include lower risk of hypoglycemia, cardio-renal protection, reduction in the number of daily injections, no need for intensification of glucose self-monitoring, simple dose titration, good tolerability.

### Second generation analogues of basal insulin

There was a large consensus (statement #24; median rating 9) in considering second-generation BI analogues associated with a lower risk of hypoglycemia with respect to previous formulations. According to panelists, a large body of evidence deriving from both randomized trials and real-world data support this statement. Also, experts agreed that the lower risk of hypoglycemia associated with second-generation BI analogues allows titration aimed at more stringent metabolic control compared to previous formulations (statement #25; median rating 8), as supported by real world data. Second-generation BI analogues were also considered by the panelists as an option allowing easy and efficient titration by the patient (statement #26; median rating 8). In particular, it was underlined that the low risk of hypoglycemia provides reassurance to the patient, who feels more confident in titrating BI doses.

Experts agreed, though with a lower level of relevance (statement #27; median rating 7), that flexibility in the timing of administration of 2Bis compared to the usual timing (i.e.: ± 3 h for Gla-300 and 8–10 h range for IDeg-100 administrations) allows easier achievement of glycemic targets. In principle, flexibility in the timing of administration could facilitate titration and improve treatment adherence, particularly in patients with irregular lifestyle habits; however, some of the panelists underlined the lack of solid evidence supporting this statement. Similarly, although there was agreement regarding the concept that flexibility in the timing of administration of second-generation BI analogues allowed for better therapeutic adherence (statement #28; median rating 7.5), the consensus was mainly based on theoretical grounds and personal experience of participants. Finally, there was a large consensus (statement #29; median rating 9) that the pharmacokinetic and pharmacodynamic characteristics of second-generation BI analogues allow them to be administered at different times of the day (e.g., in the morning).

## Conclusion

Since its discovery, insulin therapy has represented a mainstay in the therapy of diabetes, including T2D, where insulin supplementation is often needed to achieve glucose control. However, the revolution in the therapeutic approach to T2D witnessed in the last decades [1, 2] imposes a rethinking of the placement of insulin therapy in this context.

This Delphi project aimed to cover several areas of uncertainty around insulin therapy in T2D, including the (1) the role of metabolic control in the era of the “treat to benefit” approach; (2) how to start basal insulin therapy (add on vs. switch) after GLP-1 RA treatment; (3) the impact of clinical inertia (to initiate and to titrate); (4) how to intensify insulin therapy with the other available injectable therapies (free vs. fixed ratio combinations with GLP-1 RAs); (5) when and how de-intensifying from a basal-bolus regimen; (6) the potential benefits of second-generation basal insulin analogues.

A total number of 29 statements has been developed and submitted to the judgment of a group of 31 Italian expert diabetologists, representative of national geographical distribution, with a response rate of 100%. The choice of including only diabetologists in the panel relays to the fact that in Italy patients on insulin therapy are largely in charge of specialistic diabetes centers.

The first results that merit to be commented on is that for none of the 29 statements, panel members showed significant disagreement; moreover, 26 statements were considered as relevant, for one item, as expected, there was agreement that it was not relevant and only for two statements the relevance was uncertain. This supports the clearness of the statements and, on the other side, that the chosen topic of questioning about insulin therapy was felt as a relevant issue by expert diabetologists.

### Role of metabolic control in the light of the most recent guidelines

The first group of statements was aimed to gather an expert consensus on the importance of targeting glucose control in the era of the “treat to benefit”. In Italy, approximately 50% of people with T2D have HbA1c above the commonly recommended target of 7.0% (53 mmol/mol) [10]. Reasons behind poor glycemic control are variegate, including a delay in BI initiation as well as a delay in its titration [11, 12]. Not surprisingly, all the panelists agreed that achieving a HbA1c value < 7% is crucial to prevent microvascular disease, and this statement receives a full support by literature data and current international and national guidelines [1, 2, 13]. Overall, panelists recognized the importance of targeting glucose control also to prevent macrovascular disease. This statement is largely supported by current literature on the beneficial effects of reducing HbA1c on cardiovascular risk [14–16], including the more recent evidence on the role of glucose control in mediating the benefits of innovative drugs in the cardiovascular outcome trials (CVOTs) [17]. Moreover, panelists underlined the importance of an early achievement of glucose targets during the time course of diabetes, because of the legacy effect phenomenon, which is well documented also in more recent studies [18]. However, consensus on macrovascular disease was mitigated, likely because of the results of large CVOTs [19–21], by the cardiovascular consequences of hypoglycemia, which is the counterbalance of tight glucose control, and of the overall assumption that cardiovascular risk is multifactorial and needs to be addressed by an integrated approach, not limited to correcting hyperglycemia [22].

Panelists agreed that 2nd-generation BI analogues are able to achieve a similar reduction in HbA1c levels, paying a milder tribute to hypoglycemia, when compared to 1st generation BI analogues, as well demonstrated by RCTs [23]; in line with the lower hypoglycemic risk associated to the use of 2nd-generation BI analogues, panelists agreed that more stringent targets can be safely achieved by many patients treated with this class of drugs.

### Basal insulin intensification strategies: add-on versus switch

The second issue examined by panelists related to how to start BI therapy, comparing *pros and cons* of add-on versus switch of insulin therapy in patients already treated according to modern guidelines. Indeed, current guidelines [1, 13] recommend a GLP-1 RA as the first injectable therapy in T2D, followed by BI when needed, but strategies on how to start insulin therapy are still questionable. Notably, the essential role of insulin therapy in cases of hypercatabolic states and other specific clinical situations received full support by panelists, as well as by current guidelines [1]. Besides these selected cases, a large body of evidence supports the add-on strategy to achieve glucose targets, in order to maintain the cardiorenal protection offered by GLP-1 RAs or SGLT2i [24, 25].

The multiple beneficial effects of GLP-1 RAs are widely recognized by the most recent ADA/EASD and Italian guidelines [1, 2, 13] recommending a GLP-1 RA as a first line injectable agent, independently from HbA1c level and/or body weight. Therefore, GLP-1 RA should be continued even in the absence of the HbA1c or body weight target achievement, because of the expected beneficial cardio-renal effects. However, in real life, most patients pre-treated with GLP-1 RA switch to, instead of adding on BI to ongoing GLP-1 RA therapy. It has been reported that about 40% of insulin naïve T2D adult patients treated with GLP-1 RA introduce BI in their intensification strategy, and the switch is the most common approach [26]. Similarly, in Italy, the RESTORE study showed that 67.6% of insulin naïve patients with T2D on GLP-1 RA who need to intensifying therapy switched to BI (22.1% also starting 1–3 injections of short-acting analogues), 22.7% added BI while maintaining GLP-1 RA, and 9.7% switched to FRC, although effectiveness was improved with the add-on schemes [4]. Thus, while advantages of combination therapy of BI with GLP-1 RA and SGLT2i are well documented, the switch of ongoing GLP-1 RA to BI approach seems to be limited to cases of contraindications, tolerability issues, or unwanted weight loss. In this regard, in a large cohort of insulin naiveT2D patients on GLP-1 RA, the earlier addition of insulin was associated with better glycemic control, while switching to insulin was less beneficial, suggesting that in patients no longer responding to GLP-1 RA, a greater benefit would come from adding rather than switching to insulin therapy [27].

### Basal insulin intensification strategies: inertia to initiate and inertia to titrate

Clinical inertia has been recognized as another important issue by panelists of this Delphi project, being insulin therapy often delayed and under titrated. A retrospective, observational study from US and UK databases showed that in T2D patients inadequately controlled on oral glucose-lowering drugs, initiation of first injectable therapy (GLP1 RA or BI) did not occur until HbA1c was considerably above target, when control was harder to achieve [28]. Notably, addition and/or switching to more potent therapies in T2D patients not well controlled on either injectable therapy should be considered within one year, to reach glucose targets [28]. However, up-to one third of T2D patients needing therapeutic intensification undergo clinical inertia, with a large impact on clinical outcomes [29]. A recent analysis from the large AMD Annals Initiative database, by using the Artificial Intelligence approach, identified clinical drivers of inertia to start insulin therapy, focusing on the role of “tolerant waiting,” i.e., patients with borderline high HbA1c levels or showing an HbA1c increase < 0.6% between two consecutive visits are those experimenting the longest inertia [30].

Panelists were also concord on the need to timely titrate BI, and that under-titration may contribute to treatment failure [31, 32]. Also, there was a strong agreement that self-titration of BI should be always recommended, except in that minority of patients who are not able to manage it, although the role of caregivers has been emphasized in these cases. Thus, self-titration (including caregivers) was considered an efficacious approach to reach glucose targets, as demonstrated by the Italian Titration Approach Study (ITAS), showing comparable HbA1c reductions and similarly low hypoglycemic risk in poorly controlled, insulin-naïveT2D patients who initiated self- or physician-titrated Gla-300 [33], also confirmed across a range of phenotypes [34].

### Intensification strategies: free versus fixed ratio combination

There was uncertainty as to whether BI should be added to the GLP-1 RA only after the maximum tolerated dose of GLP-1 RA has been reached. Panelists recognized that free combinations have the advantage of potentially attain the maximum dosage of GLP-1 RA, thus maximizing its beneficial “dose-dependent” effects on cardiovascular protection as well as on glucose and body weight [35, 36]. Moreover, the free combination reduced the risk of over-basalization, lowering insulin units needed to optimize glucose control [37]. On the other hand, it was commented that often BI is introduced in free combination before reaching the maximum tolerated dose of GLP-1 RA, particularly in case of poor metabolic control.

Conversely, panelists agreed, although with a lower level of relevance, that FRC usually do not allow to reach the maximum dosage of GLP-1 RA, thus potentially limiting extraglycaemic effects. Notably, panelists commented that this statement should apply to cardiovascular protection and body weight, while it is less consistent for the impact on other risk factors, such as lipid profile, as demonstrated in the LixiLan-L study [38].

Agreement was also reached relative to the lower risk of hypoglycemia, weight control and simplicity of administration of FRCs, potentially improving adherence, while it was highly debated whether FRCs would mitigate side effects of GLP-1 RA, and the corresponding statement was considered uncertain [39, 40]. In fact, according to some panelists, the advantage lies mainly in the low dose of GLP-1 RA, that would be better tolerated, in favor of a more consistent BI support, with a synergistic action on glucose targets. However, according to others, the major limitation of this approach is represented by the difficulty in reaching an adequate dosage of one or the two molecules.

### Intensification strategies and de-intensification from basal-bolus in type 2 diabetes

Panelists agreed also on the benefits of an intensification approach by adding GLP-1 RA to BI, in fixed or free combination, with the advantages of the extra-glycemic benefits of GLP-1 RA, as documented by several studies, such as the Get-Goal [41, 42], Lixilan [43–45] and Dual [46–50] clinical programs.

Moreover, a strong consensus was reached regarding the opportunity to periodically reassess the effective need for insulin basal-bolus therapy, considering the lack of de-prescription as the other face of clinical inertia. In this regard, panelists discussed on the modalities of de-prescribing basal-bolus insulin therapy. Real world data showed that withdrawal of pre-prandial insulin is feasible in about 50% of T2D patients, especially those with a better residual beta-cell function (younger, with shorter disease duration, lower HbA1c, and needing lower insulin doses) [51]. Panelists agreed that the addition of GLP-1 RA can allow a suspension of prandial boluses while maintaining glycemic control and improving quality of life, as supported by a consistent literature [37]. Panelists also convened that FRCs can represent a strategy to improve simplification and consequent de-intensification from a basal-bolus insulin regimen. This approach is supported by the results of the BEYOND trial [8], showing that it is possible and safe to switch from a basal-bolus regimen to a once-daily fixed-combination injection added to BI, with similar glucose control, fewer insulin doses, fewer injections daily, and less hypoglycemia.

### Second-generation analogues of basal insulin

Panelists showed a large consensus regarding the lower risk of hypoglycemia demonstrated for 2BIs [6], and for its practical consequences, including an easier titration, self-titration, and the achievement of more ambitious targets [52]. Panel consensus on these aspects is also in line with the Italian guidelines [13], recommending the use of 2BIs for all patients with type 2 diabetes who require BI. It remains to be established whether Gla-300 and IDeg present different efficacy and safety profiles in specific subgroups of patients. Additional studies are needed to address this issue.

Also, experts agreed on the importance of flexibility in the timing of administration, and its impact on adherence, although these statements were less supported by current literature. The differences in pharmacokinetic and pharmacodynamic properties and their impact on flexibility have been fully recognized.

Our study has some limitations. First, even if consensus was reached, results were dependent on the composition of the panel. However, to minimize the potential for selection bias, panelists were selected for their long-lasting experience in diabetology and their nationwide distribution. Furthermore, all the invited experts participated in the project, thereby ensuring that the range of expert opinion was adequately represented, and consensus was obtained following a standard procedure, defined a priori.

Second, the panel was composed only by diabetes specialists, and primary care physicians were not involved. Nevertheless, given the large number of diabetes clinics in Italy and their homogeneous geographic distribution, the referral of patients needing insulin therapy to specialist care is common practice.

In conclusion, insulin therapy still represents an important strategy to achieve stringent glucose targets in order to prevent micro- and macrovascular complications in T2D patients. Besides specific circumstances in which it is essential, BI is to be promptly prescribed when GLP-1 RA or oral agents fail to maintain glucose control, thus avoiding clinical inertia. As for the modalities to prescribe insulin therapy, the add-on to existing therapy with innovative drugs offers the advantages of maintaining cardiorenal protection, while targeting glucose control. Free combination is advantageous for the possibility to modulate GLP-1 RA dose in order to implement cardiorenal effectiveness, while FRC may be implemented in some patients in order to simplify treatment and increase adherence. The need of insulin therapy as well as its regimens should be regularly revisited, opting for therapies capable of increasing adherence, even if with the same effectiveness as others, often including a GLP-1 RA, when possible. Overall, 2BI appear to be one of the preferred treatment options for their pharmacological profile and self-titration and flexibility of administration that may increase adherence and improve outcomes.

## Data Availability

Not applicable.

## Abbreviations

1BI: First-generation analogue of basal insulin
2BI: Second-generation analogue of basal insulins
ADA: American Diabetes Association
AMD: Associazione Medici Diabetologi
BI: Basal insulin
CVOT: Cardiovascular Outcome Trial
EASD: European Association for the Study of Diabetes
FRC: Fixed-ratio combination
Gla-300: Glargine 300 U/mL
GLP-1 RA: Glucagon-like peptide-1 receptor agonists
HbA1c: Glycated hemoglobin
iDeg: Degludec 100 U/mL
IPR: Interpercentile range
IPRAS: Interpercentile range adjusted for symmetry
RCT: Randomized clinical trials
SGLT2i: Sodium–glucose cotransporter-2 inhibitors
SID: Società Italiana di Diabetologia
T2D: Type 2 diabetes mellitus
